# Induced swimming reduced stress and modulated immune response and antioxidant status in juvenile rainbow trout (*Oncorhynchus mykiss*)

**DOI:** 10.1007/s10695-025-01569-w

**Published:** 2025-08-28

**Authors:** Carlos Espírito-Santo, Carmen Alburquerque, Thaís Cavalheri, Francisco A. Guardiola, Rodrigo O. A. Ozório, Leonardo J. Magnoni

**Affiliations:** 1 FCUP, Faculty of Sciences, University of Porto, Porto, 4169-007 Portugal; 2 CIIMAR/CIMAR-LA, Interdisciplinary Centre of Marine and Environmental Research, University of Porto, Matosinhos, 4450-208 Portugal; 3 Immunobiology for Aquaculture Group, Department of Cell Biology and Histology, Faculty of Biology, University of Murcia, Murcia, 30100 Spain; 4 The New Zealand Institute for Plant and Food Research Limited, Nelson, 7043 New Zealand

**Keywords:** Swimming, Stress response, Immune response, Oxidative stress, Fish welfare

## Abstract

Promoting swimming in aquaculture has been proposed to enhance fish welfare. This study investigated the effects of 6 h of swimming on stress, immune, metabolic, and antioxidant status of juvenile rainbow trout (*Oncorhynchus mykiss*). Fish (*n* = 8) were individually subjected to one of four experimental conditions: minimal water flow (control, < 0.1 body-lengths (BL)·s⁻^1^); low-speed (L, 0.8 BL·s⁻^1^); high-speed (H, 2.3 BL·s⁻^1^); and oscillating speeds (O, 0.8/2.3 BL·s⁻^1^). Plasma cortisol levels were lower in swimming groups (L, H, O) than that in the control group, indicating reduced stress. Lysozyme and peroxidase activities increased in skin mucus of fish from L and H groups, reflecting enhanced mucosal immunity. Expression of cytokines (*tnfα*, *il1β*), inflammation modulators (*nfκb1*), and immune-related (*lyz*, *ctsd*) genes in head-kidney, gills, and heart did not vary among groups. In red muscle, increased citrate synthase activity across swimming conditions reflected enhanced aerobic metabolism, while cytochrome c oxidase activity was higher in fish from H and O groups, indicating elevated mitochondrial oxidative capacity. Lactate dehydrogenase activity was higher in red muscle in the O group, reflecting greater reliance on anaerobic metabolism under oscillating swimming. Increased GSH/GSSG ratio in red and white muscle of L and H groups indicated improved antioxidant status. Nonetheless, higher lipid peroxidation levels in the liver suggest increased oxidative stress in the O group, indicating that oscillating swimming may impose metabolic and oxidative challenges. Results show that short-term induced steady swimming under L and H conditions could be used beforehand when applying procedures that decrease welfare in farmed rainbow trout, such as handling or transport, aiming to reduce stress, improving immune and antioxidant responses.

**Supplementary Information:**

The online version contains supplementary material available at 10.1007/s10695-025-01569-w.

## Introduction

Most fish species spend the majority of their life continuously swimming in their natural environments, in which they often experience changing types of water flow conditions (Liao 2007). The ability of fish to sustain different levels of swimming activity over extended periods of time, while conserving energy, has been under increased focus in fish research in recent years (McKenzie et al. 2021; Liu et al. 2023). Early studies highlighted the potential of induced swimming as a method for fostering body growth, while minimizing stress and improving both nutrient use and the overall welfare of fish (Young and Chech, 1993; Davison and Herbert 2013; Huntingford and Kadri 2013). It has been described that moderate, continuous (steady) swimming can reduce the energy expenditure related to negative behavioral interactions, such as aggression, allowing a more efficient energy allocation toward growth (East and Magnan 1987; Jobling et al. 1993; Steinhausen et al. 2010). Interestingly, recent studies have investigated the potential modulation of several welfare indicators such as innate immune parameters (Zhu et al. 2016; Liu et al. 2018; Espírito-Santo et al. 2023, 2024b) and oxidative stress biomarkers (Aniagu et al. 2006; Amérand et al. 2017; Ceccotti et al. 2019; Sánchez-Moya et al. 2022; Espírito-Santo et al. 2024a) by different induced swimming conditions, with the results being highly variable depending on the species, development stage, and condition applied.

The ability of fish to swim against different water currents through a combination of fin movements and flexion of several body segments, minimizing energy requirements, is particularly evident in salmonids, a very active fish group with excellent sustained and sprinting swimming capacities (Pakkasmaa and Piironen 2000; Milner et al. 2012). Several studies have investigated the physiological effects of swimming activity in salmonids, particularly in modulating growth rate and feed intake (Davison and Goldspink 1977; Christiansen and Jobling 1990). Interestingly, some studies have shown that certain swimming conditions may have beneficial effects on the health and welfare status, particularly in Atlantic salmon (*Salmo salar*). For example, Castro et al. (2011) found that an aerobic swim training regimen of 6 weeks enhanced Atlantic salmon resistance to the infectious pancreatic necrosis virus, accompanied by down-regulation of pro-inflammatory cytokine genes in cardiac muscle. In addition, Boesgaard et al. (1993) and Herbert et al. (2011) showed that restraining swimming activity in Atlantic salmon resulted in an increase in stress responses. Nevertheless, the relation between induced swimming activity and welfare parameters (e.g., immune and antioxidant status) remains to be elucidated in rainbow trout (*Oncorhynchus mykiss*).

Rainbow trout is a salmonid species, intensively produced worldwide due to its high adaptability to different environmental conditions, with an important role in recreational fishing and commercial farming (Bobe et al. 2016). The swimming physiology of rainbow trout has been extensively studied, with valuable insights regarding the effects of swimming activity on energy use (Magnoni et al. 2008), muscle development (Magnoni et al. 2013), and sexual maturation (Palstra et al. 2010). Interestingly, rainbow trout have been described to reduce circulating cortisol levels upon induction of swimming activity (Woodward and Smith 1985), although such a response is greatly dependent on the development stage and swimming condition applied (McKenzie et al. 2012). Still, the modulation of the immune and antioxidant status of rainbow trout by induction of swimming activity remains to be elucidated.

In aquaculture conditions, confinement in intensive rearing conditions, sub-optimal water flow velocity, and/or rearing densities may result in changes in the natural swimming behavior and stress responses (Ashley 2007). These changes are particularly evident in salmonids that usually face higher water flow rates in their natural habitats, adjusting their swimming activity accordingly (Palstra and Planas 2011).

The use of raceways is an effective system to promote swimming activity (Fornshell, et al., 2012), only when well-adjusted for the requirements of the fish species. Therefore, the implementation of swimming regimens in commercial aquaculture farms may be a useful tool to improve fish performance, resistance to disease outbreaks, and wellbeing. In addition, short-term induced swimming protocols could also be beneficial to prepare fish in advance for stressful interventions involved in farming, such as handling and transport, aiming to improve physiological responses. Therefore, this study investigated the application of 6 h of several induced swimming conditions in rainbow trout to evaluate the mechanisms regulating the physiological effects of exercise on immune function and oxidative stress, drawing from similar research conducted in mammals (Yang et al. 2005; Bessa et al. 2016; Nieman and Wentz 2019; Espírito-Santo et al. 2023, 2024a, 2024b). Furthermore, the use of a short-term swimming protocol allows the establishment of an experimental framework while minimizing confounding factors, such as feeding or social interactions. Thus, the present study aimed to assess the impact of short-term induced swimming at various water flow speeds on the metabolic response and welfare indicators in rainbow trout. The parameters were chosen for their roles in mediating the physiological response to physical activity: metabolic and stress markers in plasma, innate immune parameters in plasma and skin mucus, enzymatic activities related to aerobic and anaerobic metabolism in red and white muscle, and oxidative stress biomarkers in muscle and liver. Gene expression of key immune and inflammatory regulators was also evaluated in highly relevant tissues to explore potential transcriptional modulation.

## Materials and methods

### Fish rearing conditions

Juvenile rainbow trout were supplied by Truticultura do Minho (Paredes de Coura, Portugal) and transported to the Bioterium of Aquatic Organisms (BOGA) of CIIMAR (Interdisciplinary Centre of Marine and Environmental Research of the University of Porto). During this period, fish were fed twice a day with a commercial diet (Table S1, Supplementary Information). Afterward, 40 juvenile trout (body length: 13.7 ± 0.4 cm; body weight: 36.3 ± 2.4 g, mean ± SEM) were randomly distributed in 5 tanks (210 L, 8 fish per tank) connected to a recirculating aquaculture system (RAS) under controlled conditions and daily monitored (16 ± 1 °C; 0.3 ± 0.1‰; pH 6.0 ± 0.5; > 95% air saturation; 12 h:12 h photoperiod). Water ammonium and nitrite levels were below 0.05 mg·mL^−1^ and 0.5 mg·mL^−1^, respectively. Fish were maintained under these conditions for 6 weeks until the start of the experiment.

### Evaluation of the critical swimming speed

Before the swimming trials, the critical swimming speed (*U*_*crit*_) was determined using eight juvenile trout, according to the method described by Brett (1964), employing an adaptation of Brett-type swimming flumes. The swimming flume was submerged into a 300 L ambient tank and consisted of an acrylic cylindrical tube (length: 50 cm; inner diameter 8.5 cm) connected to an electronically controlled propeller (EcoDrift 20.1, AquaMedic, Bissendorf, Germany) fitting at one end of the flume, which generated a unidirectional water current. Water passed through a honeycomb flow rectifier to ensure a uniform flow inside the swimming flume. A grid at the end of the chamber prevented fish from being carried out of the flume. Eight fish were individually acclimatized overnight at near null speed (< 1 cm·s^−1^). Fish were not fed during this time. On the following day, the speed was increased in steps of 10 cm·s^−1^ every 15 min until the fish reached exhaustion. This condition was defined as when the fish was unable to continue swimming and remained at the back of the grid for at least 10 s. The *U*_*crit*_ was calculated according to Brett (1964):

$${U}_{crit}={v}_{f}+{v}_{i}*({~}^{{t}_{f}}\!\left/ \!{~}_{{t}_{i}}\right.)$$

*v*_*f*_ is the highest speed maintained for an entire interval; *v*_*i*_ is speed increment; *t*_*f*_ is the time the fish swam until exhaustion at the final speed; *t*_*i*_ is the time increment.

The *U*_*crit*_ was found to be 59.9 ± 4.0 cm·s^−1^ (mean ± SEM, *n* = 8), which was converted to BL·s^−1^ by dividing the swimming speed by the total body lengths of fish (4.61 ± 0.3 BL·s^−1^). Fish used in the *U*_*crit*_ trials were not used in further experiments to avoid any potential confounding effects of prior exhaustive swimming on the physiological parameters evaluated.

### Induced swimming experiment

A similar adaptation of the Brett-type swimming flumes used for the *Ucrit* assessments was employed to induce swimming during the experiments. Thirty-two fish (body length: 13.0 ± 0.2 cm; body weight: 30.14 ± 2.3 g) were transferred from the holding tanks and were individually subjected to 6 h of different experimental conditions, assessing 8 fish per condition as follows: low (L, 0.8 BL·s−1); high (H, 2.3 BL·s−1); oscillating (O, 0.8/2.3 BL·s−1) swimming speeds, and a control group not induced to swim, with minimal water flow inside the flume (control, < 0.1 BL·s−1). Therefore, swimming speeds used for these experimental conditions varied between 0.8 and 2.3 BL·s−1, representing between 17 and 50% of the *Ucrit* value calculated as described before. In the O group, each speed oscillation cycle lasted 16 seconds, following a sinusoidal wave pattern, with gradual speed increments and decreases of 5 cm⋅s−1 every 2 s. The water flow oscillated between 5 cm⋅s−1 (valley) and 30 cm⋅s−1 (peak), corresponding to a speed range of 0.8 to 2.3 BL·s−1. Each fish was transferred from a holding tank to a swim flume 16 h before the trials for acclimatization and was not fed during this period. The experiment was conducted over 4 consecutive days, using all the fish from one holding tank each day (4 tanks in total, 8 fish per tank). On each day, fish from the selected tank were randomly assigned to one of the four swimming treatments (*n* = 2 fish per condition). Fish in the fifth tank (*n* = 8) were used solely for the *Ucrit* determinations. This approach ensured that each tank was sampled only once, avoiding repeated handling and disturbance across days. Fish were individually placed in flumes and randomly assigned to four treatments the day before testing to reduce systematic bias. The initiation of swimming trials was done according to a pre-determined schedule, allowing sampling at the end of the 6 h exposure without overlapping between treatments. At the end of the swimming trials, fish were carefully and quickly removed from the swimming flumes and anesthetized in 3-aminobenzoic acid ethyl ester (MS-222; 0.1 g·L−1) buffered with NaHCO3 (0.2 g·L−1). All handling, induction, and sampling procedures were standardized and carried out to ensure consistency.

### Tissue sampling

Firstly, skin mucus was obtained by gently scraping the dorsolateral surface of the fish using a cell scraper, ensuring no contamination from blood, urogenital, or excretions, according to Guardiola et al. (2014). The skin mucus was centrifuged (2000 × *g*, 10 min, 4 °C), and the supernatants were stored at − 20 °C until further use. Afterward, blood samples were drawn from the caudal vessels using heparinized syringes, with one aliquot used to measure hematological parameters, while the remaining blood was centrifuged (10,000 × *g*, 5 min, 4 °C) to separate the plasma, which was stored at − 20 °C. The fish were then euthanized by cutting the spinal cord. Head-kidney, gills, and heart were collected for gene expression analysis and stored at − 80 °C. Red and white skeletal muscle, from the caudal and dorsal-anterior regions, respectively, and liver were collected for enzymatic activities assays.

### Hematological parameters

The percentage of hematocrit in blood was assessed by centrifuging blood in micro-hematocrit capillary tubes (10,000 × *g*, 5 min). The hemoglobin levels were measured by the cyanomethaemoglobin method using a commercial kit (SPINREACT, Girona, Spain). In short, 10 μL of blood were diluted in 2.5 mL of Drabkin’s solution. The absorbance of the sample was measured at 540 nm using a microplate reader (BioTek Instruments, Winooski, USA) at room temperature. A hemoglobin standard of known concentration (15 g·dL^−1^) was used to calculate the hemoglobin concentration in the samples.

### Stress and energy mobilization markers in plasma

Glucose and lactate levels in plasma were measured using commercial enzymatic colorimetric assay kits (GOD-POD and LO-POD, SPINREACT, Girona, Spain). The glucose assay was based on the enzymatic reaction catalyzed by glucose oxidase, which oxidizes glucose to gluconic acid. During this reaction, hydrogen peroxide (H₂O₂) is produced and subsequently detected using a chromogenic oxygen acceptor in the presence of peroxidase. In brief, 10 µL of sample were incubated in 1 mL of the reaction solution. The absorbance of the samples was measured at 505 nm using a microplate reader (BioTek Instruments, Winooski, USA) at room temperature. A glucose sample of known concentration (100 mg·dL^−1^) was used to determine the glucose concentration in each plasma sample. The lactate determination was based on the enzymatic reaction catalyzed by lactate oxidase, which oxidizes lactate to pyruvate and H₂O₂. The H₂O₂ reacts with 4-aminophenazone and 4-chlorophenol in the presence of peroxidase. In brief, 10 µL of plasma were incubated in 1 mL of the reaction solution and the absorbance was read at 505 nm using a microplate reader (BioTek Instruments, Winooski, USA) at room temperature. A lactate standard of known concentration (10 mg·dL^−1^) was used to determine the lactate concentration in each plasma sample. Plasma cortisol concentration was determined with an enzyme immunoassay kit based on the competitive binding between cortisol and specific monoclonal antibodies (RE 52061, IBL International, Hamburg, Germany). A standard curve using seven samples of known concentrations was performed to calculate the concentration of cortisol in each sample. The absorbance was measured at 450 nm at room temperature using a microplate reader (BioTek Instruments, Winooski, USA). This particular kit was previously used and validated for cortisol concentration measurements in the plasma of rainbow trout (Magnoni et al. 2019). All assays were conducted in triplicate.

### Innate immune parameters in skin mucus and plasma

The activity of the alternative complement pathway in plasma was determined following the method described by Sunyer and Tort (1995). Three buffers were prepared: isotonic veronal-buffered saline (GVB; pH 7.3; 0.1% gelatine); EDTA-GVB, as the previous buffer but containing 20 mM EDTA; and Mg-EGTA-GVB, consisting of GVB with 10 mM Mg^2+^ and 10 mM EGTA. Subsequently, rabbit red blood cells (RaRBC; Probiologica Lda.) were washed four times in GVB buffer and resuspended in the same buffer to a concentration of 1.93 × 10^8^ cells·mL^−1^. Then, 10 μL of RaRBC suspension was added to 40 μL of serially diluted plasma samples in Mg-EGTA-GVB buffer. After that, plasma samples were incubated at room temperature for 120 min with constant shaking. Afterwards, 150 μL of EDTA-GVB buffer was added to stop the reaction. Lastly, the samples were centrifuged (120 × *g*, 2.5 min) and the extent of hemolysis was calculated by assessing the absorbance of the supernatants at 414 nm in a microplate reader (BioTek Instruments, Winooski, USA) at room temperature. The ACH_50_ units were defined as the plasma concentration that induces 50% hemolysis of RaRBC.

Lysozyme activity in both plasma and skin mucus was determined through a turbidimetric method described by Swain et al. (2007). In brief, 20 μL of plasma or skin mucus were added to 96-well flat-bottomed plates and, to each well, 180 μL of freeze-dried *Micrococcus lysodeikticus* (0.2 mg·mL^−1^; Sigma-Aldrich, Hamburg, Germany) in 40 mM sodium phosphate (PBS; pH 6.2) was added to serve as lysozyme substrate. In addition, to act as a blank for each sample, 20 μL of plasma or skin mucus was added to 180 μL of PBS. The absorbance at 450 nm was assessed after an incubation of 20 min at 35 °C. The amount of lysozyme in plasma and skin mucus was determined using a standard curve prepared with hen egg white lysozyme (HEWL; Sigma-Aldrich, Hamburg, Germany) through serial dilutions in sodium phosphate buffer. The activity of lysozyme was expressed as U·mL^−1^ equivalent of HEWL activity.

Peroxidase activity in plasma and skin mucus was determined following the protocol of Quade and Roth (1997). In brief, 15 μL of plasma or 30 μL of skin mucus were diluted with 135 or 120 μL of Hank’s buffer (HBSS) without Ca^2+^ or Mg^2+^, respectively. Afterward, 50 μL of 3,3′,5,5′-tetramethylbenzidine hydrochloride (TMB, 20 mM) and H_2_O_2_ (5 mM) were added to act as substrates. The reaction was stopped following 2 min through the addition of H_2_SO_4_ (2 M). The absorbance was measured at 450 nm at 25 °C. Standard samples without plasma or skin mucus were used to serve as blanks. A unit of peroxidase activity was defined as a change in absorbance and expressed per mL of sample.

Protease activity in plasma and skin mucus was determined following the protocol by Magnoni et al. (2023). Briefly, 30 µL of plasma or skin mucus diluted (1:3) with PBS (115 mM; pH 7.0) was incubated for 24 h at 25 °C with 2% azocasein (Sigma-Aldrich, Hamburg, Germany). The reaction was ceased through the addition of 250 µL of trichloroacetic acid (TCA; 4.6%). After that, the mixture was incubated for 30 min at 25 °C and centrifuged for 10 min at 10,000 × *g*. Then, aliquots of 100 µL of the supernatant were transferred to each well containing 100 µL of NaOH (1 N), in triplicate. The absorbance was read at 450 nm at 25 °C. Samples were replaced by PBS and 10 µL of trypsin (5 mg·mL^−1^, Sigma-Aldrich, Hamburg, Germany) or only PBS to evaluate 100% and 0% of protease activity, respectively.

Antiprotease activity in plasma and skin mucus was assessed according to Magnoni et al. (2023). Samples previously diluted (1:3) with PBS (115 mM and pH 7.0) were incubated for 10 min at 25 °C with 10 µL of trypsin (5 mg·mL^−1^; Sigma-Aldrich, Hamburg, Germany). After that, 80 µL of PBS and 125 µL of azocasein (Sigma-Aldrich, Hamburg, Germany) were added and incubated for 1 h at 22 °C. The reaction was ended through the addition of 250 µL TCA (4.6%). The mixture was incubated for 30 min at 25 °C and centrifuged for 10 min at 10,000 × *g*. After that, 100 µL of the supernatant was transferred along with 100 µL of NaOH (1 N) to each well of a 96-well flat-bottomed plate, in triplicate. The absorbance was read at 450 nm at 25 °C. The sample was replaced by PBS in the positive controls (representing 100% protease), and both the sample and trypsin were replaced by PBS in negative controls (representing 0% protease). The results were expressed as the percentage of inhibition of trypsin activity.

### Gene expression

The expression of the selected genes (Table 1) was analyzed by real-time qPCR with QuantStudio™ Real-Time PCR System Fast (Life Technologies). Firstly, total RNA from the head-kidney, gills, and heart was extracted and purified using TRIzol™, following the manufacturer’s instructions. Quantification and purity of RNA were assessed using a Nanodrop® spectrophotometer. For the gene expression analyses, the RNA was treated with DNase I (Promega) to eliminate genomic DNA contamination, and complementary DNA (cDNA) was synthesized using the reverse transcriptase enzyme SuperScriptIV (Life Technologies) with an oligo-dT_18_ primer. The reaction mixtures containing SYBR Green Supermix, primer, and cDNA template were incubated for 10 min at 95 °C, followed by 40 cycles of 15 s at 95 °C, 1 min at 60 °C, and 15 s at 95 °C. The gene expression was analyzed using the 2^−ΔCt^ method (Livak and Schmittgen 2001). The specificity of the reactions was analyzed using samples without cDNA as negative controls and for each gene. Gene expression was normalized with the expression of elongation factor 1-alpha (*ef1α*) in each sample. The efficiency of the primers was evaluated by melt curves in which the values were within the acceptable range of 90 to 110%.

**Table 1:** Primers used for real-time qPCR

Gene name	Gene abbreviation	Accession number	Primer sequences (5′ → 3′)	Reference
**Elongation factor 1-alpha**	*ef1α*	NM_001124339.1	F: GGCAAGTCAACCACCACAG R: GATACCACGCTCCCTCTCAG	Machado et al. (2021)
**Tumor necrosis factor-alpha**	*tnfα*	NM_001124374.1	F: GGGGACAAACTGTGGACTGA R: GAAGTTCTTGCCCTGCTCTG	Han et al. (2023)
**Interleukin 1 beta**	*il1β*	AJ278242.2	F: CGTCACTGACTCTGAGAACAAGT R: TGGCGTGCAGCTCCATAG	Machado et al. (2021)
**Nuclear factor kappa subunit 1**	*nfkb1*	XM_021614113.1	F: AGCAACCAAACATCCCACCA R: CTTGTCGTGCCTGCTTTCAC	Randazzo et al. (2021)
**Lysozyme**	*lyz*	XM_021601582.2	F: GAAACAGCCTGCCCAACT R: GTCCAACACCACACGCTT	Han et al. (2023)
**Cathepsin D**	*ctsd*	NM_001124711.1	F: GCCTGTCATCACATTCAACCT R: CCACTCAGGCAGATGGTCTTA	Abolfathi et al. (2020)

### Enzymatic activities in skeletal muscle and liver

#### Tissue homogenization

Frozen samples of red and white skeletal muscle were powdered using a pestle and mortar cooled in liquid nitrogen and divided into aliquots for metabolic and oxidative stress enzyme analyses. For metabolic enzymes, samples of red and white skeletal muscle were weighed and homogenized in 20 volumes of extraction buffer (20.0 mM HEPES; 1.0 mM EDTA; 0.1% Triton X-100; pH 7.0) as described by Espírito-Santo et al. (2024a). For oxidative stress biomarkers, samples of red and white skeletal muscle and liver were homogenized in a phosphate buffer (0.1 M KH_2_PO_4_; 0.1 M K_2_HPO_4_; pH 7.4), as described by Magnoni et al. (2023). The total protein concentrations in all samples were calculated using bovine serum albumin as a standard, according to Bradford (1976). For all enzymatic assays, changes in absorption were measured at 25 °C in a Power-Wave™ microplate spectrophotometer (BioTek Instruments, Winooski, USA). All enzymatic activities were normalized by the concentration of protein previously determined. All analyses were performed in triplicates.

#### Metabolic enzymes in skeletal muscle

The activity of cytochrome c oxidase (COX, EC: 1.9.3.1) was measured immediately after homogenization according to the protocol by McClelland et al. (2005), with some modifications. In brief, 270 µL of the assay buffer consisting of reduced cytochrome c (0.05 mM) and Tris–HCl (50 mM) was added to 30 µL of homogenate, in triplicate, in 96-well flat-bottomed plates. The absorbance was read at 550 nm.

β-Hydroxyacyl CoA dehydrogenase (HOAD, EC: 1.1.1.35) activity is calculated as described by McClelland et al. (2005). Briefly, 20 µL of sample is added to 280 µL of the assay mixture (pH 7.2) consisting of imidazole (50 mM), acetoacetyl coenzyme A (0.1 mM), and NADH (0.15 mM). The absorbance of NADH oxidation is measured at 340 nm.

Lactate dehydrogenase (LDH, EC: 1.1.1.27) activity was determined according to McClelland et al. (2005). To 275 µL of assay mixture (pH 7.0) containing HEPES (50 mM), pyruvate (1 mM), and NADH (0.15 mM), 15 µL of sample was added. The consumption rate of NADH was monitored by reading the absorbance at 340 nm.

The activity of citrate synthase (CS, EC: 2.3.3.1) was calculated as described by McClelland et al. (2005). In short, 10 µL of sample was added to a mix (pH 7.2) containing 5,5′-dithiobis-(2-nitrobenzoic acid) (DTNB; 0.1 mM), acetyl coenzyme A (0.1 mM), oxaloacetate (0.5 mM), and Tris–HCl (50 mM) in 96-well flat-bottomed plates in triplicates. Control wells without oxaloacetate were used to correct background activity. Changes in absorption were determined at 412 nm.

#### Antioxidant enzymes and oxidative stress biomarkers in skeletal muscle and liver

The activity of catalase (CAT, EC: 1.11.1.6) was determined according to Claiborne (1985). Essentially, 15 µL of sample was mixed with K-Phosphate (0.05 M; pH 7.0) and H_2_O_2_ (30%; 0.03 M). The absorbance was read at 240 nm.

Superoxide dismutase (SOD, EC: 1.15.1.1) activity was measured using a commercial assay kit (19,160−1KT-F, Sigma-Aldrich, Hamburg, Germany). This assay utilizes a water-soluble tetrazolium salt, which reacts with the superoxide anion (O₂⁻) to produce a water-soluble formazan dye. The production of the dye is inhibited by SOD, which catalyzes the dismutation of O₂⁻ into H_2_O_2_ and O_2_. Since the absorbance at 450 nm is proportional to the amount of O₂⁻, the SOD activity as an inhibition activity was quantified by measuring the decrease in the color development at 450 nm. The percentage of inhibition determined in each sample was converted to SOD activity based on the IC_50_ (50% inhibition activity of SOD) provided by the kit. One unit of SOD activity was defined as the amount of enzyme required to inhibit the reduction of the water-soluble tetrazolium salt by 50%.

The activity of glutathione reductase (GR, EC: 1.8.1.7) was calculated according to Mohandas et al. (1984). In brief, 20 µL of sample was added to a reaction mixture (pH 7.5) containing NADPH (10 mM) and DTNB (0.1 mM). The reaction was started following the addition of oxidized glutathione (GSSG; 3.25 mM). Changes in absorbance were measured at 412 nm. Glutathione peroxidase (GPx, EC: 1.11.1.9) activity was calculated by adding 15 µL of sample to a reaction mixture (pH 7.0) consisting of reduced glutathione (GSH; 4 mM), NADPH (0.8 mM) and H_2_O_2_ (0.5 mM) (Cribb et al. 1989). The oxidation of NADPH was measured at 340 nm. Glutathione-S-transferase (GST, EC: 2.5.1.18) activity was determined by adding 100 µL of sample to a reaction buffer consisting of GSH (10 mM), 1-chloro-2,4-dinitrobenzene (CDNB; 60 mM) in K-phosphate (0.1 M; pH 6.5) (Habig et al. 1974). Changes in absorbance were read at 340 nm. The levels of lipid peroxidation (LPO) were quantified using the method described by Ohkawa et al. (1979). Firstly, 150 µL of homogenate was added to 500 µL of TCA (12%) followed by the addition of 400 µL of Tris–HCl (60 mM) with diethylenetriamine-pentaacetic acid (DTPA; 0.1 mM) and 500 µL of 2-thiobarbituric acid (TBA; 0.73%). The reaction mixtures were incubated for 60 min at 100 °C. Afterwards, following centrifugation (5 min, 10,000 × *g*), the absorbance was read at 535 nm. The results of LPO levels are expressed as nmol of thiobarbituric acid reactive substances (TBARS) per g of tissue. Total glutathione and GSSG levels were measured according to the protocol by Baker et al. (1990), by adding 20 µL of sample in 200 µL of TCA to a reaction mixture containing DTNB (10 mM), NADPH (1 mM), GR 2.36 (U·ml^−1^), and sodium phosphate buffer (0.2 M; pH 7.5). To measure the GSSG concentration, elimination of free thiols present in the samples was done using 2-vinyl-pyridine, prior to the reaction (Griffith 1980). The GSH concentration was then determined by subtracting the amount of GSSG from the total glutathione levels calculated. The GSH and GSSG levels are expressed as nmol of conjugated TNB formed per min per mg of protein.

### Statistical analysis

Data are expressed as mean ± standard error of the mean (SEM). Data were analyzed using one-way ANOVA, and *P* value set for less than 0.05. When significant differences among group means were detected, Tukey’s multi-comparison test was applied as post hoc test to specify these differences. The normality of the data was tested using the Shapiro–Wilk test and the homogeneity of variance using the Levene test. Non-normally distributed data were log-transformed to perform parametric tests. If data did not meet parametric assumptions, a non-parametric Kruskal–Wallis test followed by a Dunn’s multiple comparison test was used. All statistical analyses were performed with GraphPad Prism 8.0 (GraphPad Software Inc., San Diego, USA).

## Results

### Hematological parameters

No variations were observed in the hematocrit levels or hemoglobin content of fish across experimental conditions (Table S2).

### Stress and energy mobilization markers in plasma

**Fig. 1:**
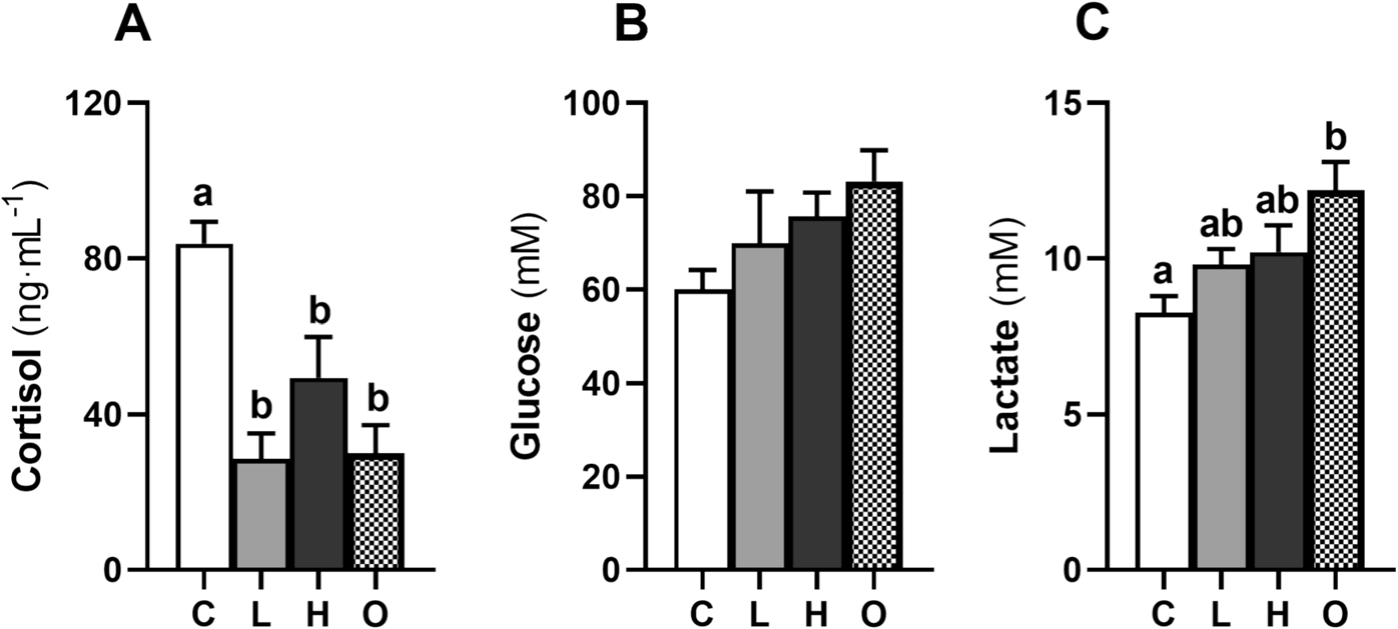
Cortisol (**A**), glucose (**B**), and lactate (**C**) concentrations in the plasma of rainbow trout (*Oncorhynchus mykiss*) subjected to different swimming conditions for 6 h: L—low-speed (0.8 BL·s⁻^1^); H—high-speed (2.3 BL·s⁻^1^); O—oscillating speeds (0.8/2.3 BL·s⁻^1^), and C—control (< 0.1 BL·s⁻^1^). The values represent mean ± SEM (*n* = 8 fish·treatment.^−1^). Different letters indicate significant variations between the experimental groups (one-way ANOVA followed by Tukey’s post hoc test, *P* < 0.05)

The concentration of plasma cortisol was lower in L (*P* < 0.001), H (*P* < 0.05), and O (*P* < 0.001) groups compared to control group (Fig. 1A). Nevertheless, plasma glucose remained unchanged in all experimental groups (Fig. 1B), while plasma lactate levels were higher in O group compared to control group (*P* < 0.01) (Fig. 1C).

### Innate immune parameters in skin mucus and plasma

**Fig. 2:**
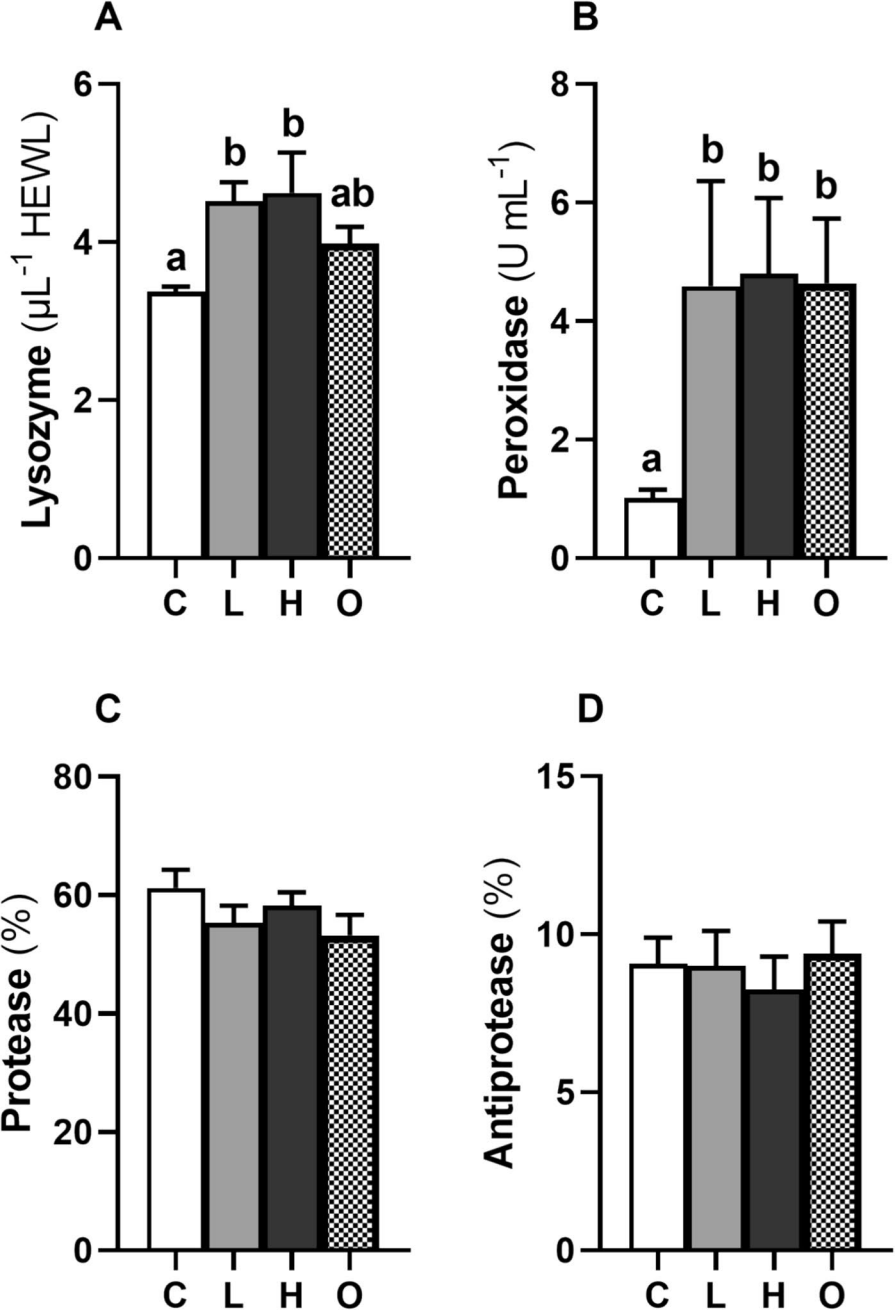
Lysozyme (**A**), peroxidase (**B**), protease (**C**), and antiprotease (**D**) activities in the skin mucus of rainbow trout (*Oncorhynchus mykiss*) subjected to different swimming conditions for 6 h: L—low-speed (0.8 BL·s⁻^1^); H—high-speed (2.3 BL·s⁻^1^); O—oscillating speeds (0.8/2.3 BL·s⁻^1^); and C—control (< 0.1 BL·s⁻^1^). The values represent mean ± SEM (*n* = 8 fish·treatment^**−**^.^1^). Different letters indicate significant variations between the experimental groups (one-way ANOVA followed by Tukey’s post hoc test, *P* < 0.05)

The activity of the alternative complement pathway, lysozyme, peroxidase, protease, and antiprotease activities quantified in the plasma of rainbow trout were not significantly different between all the experimental conditions (Table 2). However, in the skin mucus, lysozyme activity (Fig. 2A) was elevated in the L (*P* < 0.01) and H (*P* < 0.01) groups compared to the control group. Moreover, the peroxidase activity (Fig. 2B) in the skin mucus was higher in L (*P* < 0.05), H (*P* < 0.01), and O (*P* < 0.01) groups compared to control. The activities of protease (Fig. 2C) and antiprotease (Fig. 2D) in the skin mucus of trout were not significantly different between the experimental conditions applied.

**Table 2:** Innate immune parameters in plasma of rainbow trout (*Oncorhynchus mykiss*) subjected to different swimming conditions for 6 h

Innate immune parameters	Experimental conditions			
	**C**	**L**	**H**	**O**
**Alternative complement pathway** (ACH_50_·mL^−1^)	28.49 ± 3.49	29.66 ± 2.91	27.56 ± 3.11	28.07 ± 3.14
**Lysozyme** (μg·mL^−1^ HEWL)	8.33 ± 0.28	8.59 ± 0.32	8.85 ± 0.26	7.15 ± 0.86
**Peroxidase** (U·mL^−1^)	157.22 ± 30.08	145.96 ± 16.18	195.05 ± 24.71	128.59 ± 16.22
**Protease** (%)	39.77 ± 5.16	42.49 ± 4.11	42.07 ± 6.07	40.13 ± 5.54
**Antiprotease** (%)	15.19 ± 2.91	14.71 ± 3.48	15.90 ± 5.41	13.36 ± 3.87

*L*, low-speed (0.8 BL·s⁻^1^); *H*, high-speed (2.3 BL·s⁻^1^); *O*, oscillating speeds (0.8/2.3 BL·s⁻^1^); *C*, control (< 0.1 BL·s⁻^1^). The values represent mean ± SEM (*n* = 8 fish·treatment^**−**1^). No significant differences were found between groups (one-way ANOVA, *P* > 0.05)

### Gene expression in head-kidney, gills, and heart

**Fig. 3:**
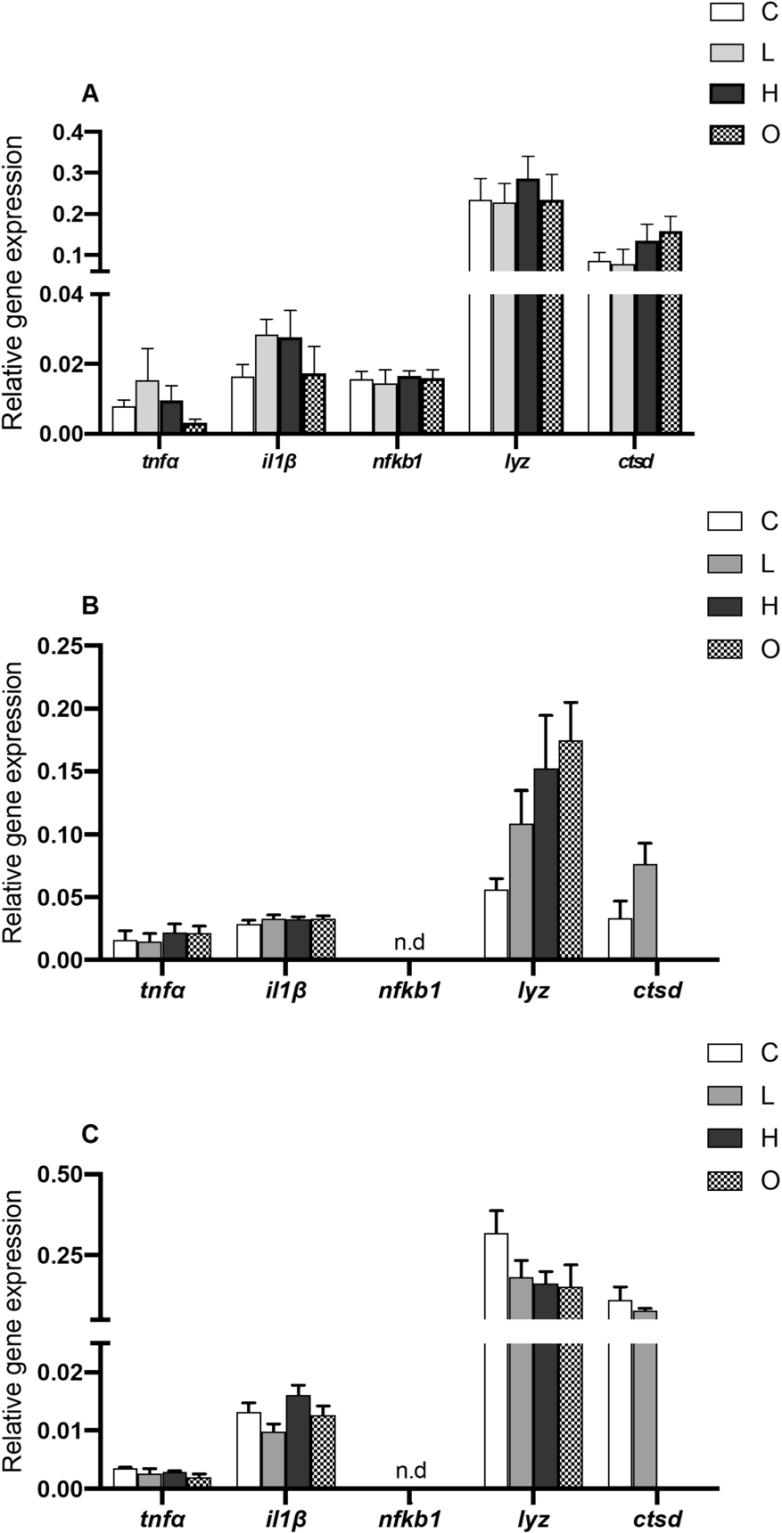
Relative gene expression of tumor necrosis factor alpha (*tnfα*), interleukin 1 beta (*il1β*), nuclear factor kappa subunit 1 (*nfκb1*), lysozyme (*lyz*), and cathepsin D (*ctsd*) in the head-kidney (**A**), gills (**B**), and heart (**C**) of rainbow trout (*Oncorhynchus mykiss*) subjected to different swimming conditions for 6 h: L—low-speed (0.8 BL·s^**−**1^); H—high-speed (2.3 BL·s⁻^1^); O—oscillating speeds (0.8/2.3 BL·s⁻^1^); and C—control (< 0.1 BL·s⁻^1^). Gene expression was analyzed by real-time qPCR and normalized to housekeeping gene elongation factor 1-alpha (*ef1α*). The values represent mean ± SEM (*n* = 8 fish·treatment^**−**^.^1^); n.d. = not detected. No significant differences were observed between groups (one-way ANOVA, *P* > 0.05)

The gene expression analysis showed no variations in the relative expression of pro-inflammatory cytokines (*tnfα* and *il1β*) and inflammation mediators (*nfκb1*) and immune-related (*lyz* and *ctsd*) genes in either the head-kidney (Fig. 3A), gills (Fig. 3B), or heart (Fig. 3C). Furthermore, no expression of *nfκb1* was detected in gills (Fig. 3B) and heart (Fig. 3C). The expression of *ctsd* was not detected in the H and O groups in gills (Fig. 3B) and heart (Fig. 3C).

### Metabolic enzymes

In red muscle, the activity of CS was significantly higher in L, H, and O (*P* < 0.05) groups when compared to the control group, while the LDH activity was only elevated in the O condition compared to the control (*P* < 0.05) (Fig. S1, Supplementary Information). The activity of HOAD did not vary between experimental groups. Additionally, the activity of COX in red muscle was higher in the H (*P* < 0.05) and O groups (*P* < 0.05) compared to C and L groups (Fig. S1, Supplementary Information). No differences were detected in the activities of CS, LDH, HOAD, and COX in white muscle of rainbow trout, regardless of the experimental conditions (Fig. S2, Supplementary Information).

### Oxidative stress biomarkers

No variations were observed in the activities of CAT, SOD, GR, and GPx in the liver of rainbow trout between experimental groups (Table 3). However, the activity of GST in the liver was higher in the H condition compared to the control (*P* < 0.05). The LPO levels were increased in the O condition compared to the control (*P* < 0.01) and the L condition (*P* < 0.01), while no variations were detected in the levels of GSH and GSSG, as well as the GSH/GSSG ratio.

**Table 3:** Activities of antioxidant enzymes, oxidative stress biomarkers in liver of rainbow trout (*Oncorhynchus mykiss*) subjected to different swimming conditions for 6 h

Antioxidant enzymes	Experimental conditions			
	**C**	**L**	**H**	**O**
**CAT** (U·mg^−1^ protein)	142.29 ± 10.74	157.63 ± 18.69	148.22 ± 14.00	144.60 ± 8.25
**SOD** (U·mg^−1^ protein)	12.09 ± 0.74	10.88 ± 0.79	11.13 ± 0.65	13.61 ± 1.94
**GR** (mU·mg^−1^ protein)	6.81 ± 0.55	7.92 ± 1.17	8.77 ± 0.81	7.34 ± 0.86
**GPx** (U·mg^−1^ protein)	45.63 ± 3.64	44.08 ± 1.94	45.52 ± 2.52	50.24 ± 2.54
**GST** (mU·mg^−1^ protein)	23.10 ± 1.94 ^a^	32.73 ± 3.07 ^ab^	35.11 ± 3.29 ^b^	28.83 ± 3.14 ^ab^
**Oxidative stress biomarkers**				
**LPO** (nmol TBARS·g^−1^ tissue)	66.24 ± 1.39 ^a^	63.96 ± 2.64 ^a^	69.78 ± 2.22 ^ab^	76.57 ± 2.60 ^b^
**GSH** (nmol TNB·min^−1^ mg^−1^ protein)	2.92 ± 0.25	3.46 ± 0.72	3.96 ± 0.65	3.71 ± 0.49
**GSSG** (nmol TNB·min^−1^ mg^−1^ protein)	1.58 ± 0.13	1.64 ± 0.20	1.55 ± 0.25	1.80 ± 0.22
**GSH/GSSG ratio**	0.96 ± 0.13	1.05 ± 0.27	1.60 ± 0.38	1.13 ± 0.23

*CAT*, catalase; *SOD*, superoxide dismutase; *GR*, glutathione reductase; *GPx*, glutathione peroxidase; *GST*, glutathione-S-transferase; *LPO*, lipid peroxidation; *GSH*, reduced glutathione; *GSSG*, oxidized glutathione

*L*, low-speed (0.8 BL·s⁻^1^); *H*, high-speed (2.3 BL·s⁻^1^); *O*, oscillating speeds (0.8/2.3 BL·s⁻^1^); *C*, control (< 0.1 BL·s⁻^1^). The values represent mean ± SEM (*n* = 8 fish·treatment^−1^). Different letters indicate significant differences between the experimental groups (one-way ANOVA followed by Tukey’s post-hoc test, *P* < 0.05)

In red muscle, no differences were observed between swimming groups on the activities of the antioxidant enzymes and the oxidative stress biomarkers LPO, GSH, and GSSG (Table 4). Nevertheless, the GSH/GSSG ratio was higher in the L and H groups (*P* < 0.05) when compared to the control group.

**Table 4:** Activities of antioxidant enzymes and oxidative stress biomarkers in red muscle of rainbow trout (*Oncorhynchus mykiss*) subjected to different swimming conditions for 6 h

Antioxidant enzymes	Experimental conditions			
	**C**	**L**	**H**	**O**
**CAT** (U·mg^**−**1^ protein)	32.86 ± 3.79	22.94 ± 2.35	22.13 ± 3.54	22.71 ± 1.71
**SOD** (U·mg^**−**1^ protein)	12.11 ± 2.03	11.08 ± 0.93	12.19 ± 1.32	11.40 ± 1.89
**GR** (mU·mg^**−**1^ protein)	5.49 ± 0.86	5.09 ± 0.58	5.12 ± 0.13	5.45 ± 0.85
**GPx** (U·mg^**−**1^ protein)	2.27 ± 0.33	2.02 ± 0.34	2.37 ± 0.95	2.37 ± 0.45
**GST** (mU·mg^**−**1^ protein)	33.69 ± 4.46	29.50 ± 3.11	40.08 ± 12.09	32.12 ± 5.45
**Oxidative stress biomarkers**				
**LPO** (nmol·TBARS·g^**−**1^ tissue)	50.60 ± 1.64	51.88 ± 2.42	50.97 ± 1.99	50.03 ± 1.75
**GSH** (nmol·TNB·min^**−**1^ mg^**−**1^ protein)	2.05 ± 0.18	2.12 ± 0.30	2.18 ± 0.19	1.92 ± 0.19
**GSSG** (nmol·TNB·min^**−**1^ mg^**−**1^ protein)	1.25 ± 0.11	0.90 ± 0.09	0.87 ± 0.09	0.92 ± 0.10
**GSH/GSSG ratio**	1.71 ± 0.09 ^a^	2.41 ± 0.11 ^b^	2.49 ± 0.13 ^b^	2.18 ± 0.16 ^ab^

*CAT*, catalase; *SOD*, superoxide dismutase; *GR*, glutathione reductase; *GPx*, glutathione peroxidase; *GST*, glutathione-S-transferase; *LPO*, lipid peroxidation; *GSH*, reduced glutathione; *GSSG*, oxidized glutathione

*L*, low-speed (0.8 BL·s⁻^1^); *H*, high-speed (2.3 BL·s⁻^1^); *O*, oscillating speeds (0.8/2.3 BL·s⁻^1^); *C*, control (< 0.1 BL·s⁻^1^). The values represent mean ± SEM (*n* = 8 fish·treatment^−1^). Different letters indicate significant variations between the experimental groups (one-way ANOVA followed by Tukey’s post hoc test, *P* < 0.05)

In white muscle, no differences were observed between experimental groups on the activities of the antioxidant enzymes, and the oxidative stress biomarkers LPO and GSH (Table 5). However, the GSSG levels in fish subjected to L and H conditions (*P* < 0.05) were lower compared to the control. Consequently, the GSH/GSSG ratio was higher in the L (*P* < 0.01) and H conditions (*P* < 0.05) compared to the control group.

**Table 5:** Activities of antioxidant enzymes and oxidative stress biomarkers in white muscle of rainbow trout (*Oncorhynchus mykiss*) subjected to different swimming conditions for 6 h

Antioxidant enzymes	Experimental conditions			
	**C**	**L**	**H**	**O**
**CAT** (U·mg^**−**1^ protein)	4.09 ± 0.66	3.90 ± 0.52	4.22 ± 0.35	4.11 ± 0.41
**SOD** (U·mg^**−**1^ protein)	8.42 ± 0.80	7.91 ± 0.86	8.01 ± 0.39	8.20 ± 0.72
**GR** (mU·mg^**−**1^ protein)	2.19 ± 0.10	2.28 ± 0.11	2.16 ± 0.20	2.16 ± 0.19
**GPx** (U·mg^**−**1^ protei**n)**	1.95 ± 0.11	1.92 ± 0.09	1.99 ± 0.10	1.98 ± 0.08
**GST** (mU·mg^**−**1^ protein)	1.52 ± 0.15	1.68 ± 0.13	1.65 ± 0.16	1.61 ± 0.13
**Oxidative stress biomarkers**				
**LPO** (nmol·TBARS·g^**−**1^ tissue)	19.91 ± 2.18	15.81 ± 3.49	16.50 ± 3.82	17.03 ± 4.55
**GSH** (nmol·TNB·min^**−**1^ mg^**−**1^ protein)	1.81 ± 0.32	2.12 ± 0.09	2.00 ± 0.11	1.95 ± 0.12
**GSSG** (nmol·TNB·min^**−**1^ mg^**−**1^ protein)	1.15 ± 0.10 ^a^	0.72 ± 0.10 ^b^	0.73 ± 0.11 ^b^	0.90 ± 0.12 ^ab^
**GSH/GSSG ratio**	1.70 ± 0.16 ^a^	2.92 ± 0.22 ^b^	2.58 ± 0.30 ^b^	2.15 ± 0.17 ^ab^

*CAT*, catalase; *SOD*, superoxide dismutase; *GR*, glutathione reductase; *GPx*, glutathione peroxidase; *GST*, glutathione-S-transferase; *LPO*, lipid peroxidation; *GSH*, reduced glutathione; *GSSG*, oxidized glutathione

*L*, low-speed (0.8 BL·s⁻^1^); *H*, high-speed (2.3 BL·s⁻^1^); *O*, oscillating speeds (0.8/2.3 BL·s⁻^1^); *C*, control (< 0.1 BL·s⁻^1^). The values represent mean ± SEM (*n* = 8 fish·treatment^−1^). Different letters indicate significant differences between the experimental groups (one-way ANOVA followed by Tukey’s post-hoc test, *P* < 0.05)

## Discussion

Rainbow trout are commonly cultured in raceways, a farming system characterized by unidirectional water current (FAO 2024). In this type of rearing setting, this species experiences consistent water flow velocities that can influence their swimming behavior, energy expenditure, and health (Welker et al. 2019). Understanding how rainbow trout respond physiologically and behaviorally to specific flow conditions is critical for optimizing health and performance that can be applied in raceways or other production systems. Furthermore, short-term induced swimming could be implemented in cultured fish as a form of preconditioning before the occurrence of stressful events programmed within aquaculture operations, such as handling and transport.

In this study, swimming speeds between 0.8 and 2.3 BL·s⁻^1^ were selected based on prior *U*_*crit*_ measurements conducted on the same population of fish, representing approximately 17% to 50% of *U*_*crit*_. These values are within the range of flow conditions reported in commercial raceways and recirculating aquaculture systems (Fornshell et al. 2012; Maillard et al., 2005; Roque d’Orbcastel et al., 2009; Parker and Barnes 2015; Royer et al. 2021). Nevertheless, *U*_*crit*_ can vary in rainbow trout and other fishes, depending on development stage, size, and environmental conditions (Kern et al. 2017; Rossi et al. 2019; Palstra et al. 2020).

It is important to note that this study was conducted under controlled laboratory conditions using individually tested fish over 6 h. While this design does not aim to replicate the group dynamics or duration of aquaculture settings, it allows for an assessment of immediate physiological responses to induced swimming. These results, therefore, aid in understanding how short periods of controlled swimming can influence stress and immune-related physiology in rainbow trout.

### Stress and immune-related responses

Plasma cortisol was measured as a primary stress marker, while glucose and lactate levels were assessed to evaluate trout responses related to energy mobilization and anaerobic metabolism. In this study, fish subjected to 6 h of swimming caused a reduction of plasma cortisol levels in all swimming groups compared to the control group. Cortisol, a key stress hormone in teleosts, is central to the primary neuroendocrine stress response, facilitating various adaptive processes such as metabolism, osmoregulation, and immunity (Gorissen and Flik 2016). Previous studies have revealed that highly efficient swimmers such as other salmonids and eels display lower plasma cortisol levels during long-term swimming migration, suggesting that these species can minimize stress responses and maximize their immune system (Totland et al. 1987; Boesgaard et al. 1993; Van Ginneken et al. 2002). Moreover, it has been suggested that restraining salmonids from swimming activity may itself be intrinsically stressful (Rodnick and Planas 2016). It is important to consider that, in this study, all fish were individually housed in flumes during the experimental period. Although an overnight acclimation period was applied in the swimming flumes before the start of the swimming trials to allow recovery from handling and to minimize the effects of a new environment on the physiological and behavioral response of trout, we cannot discard the confined setting to be a mild stressor. In this context, comparisons among fish exposed to similar housing and handling conditions but subjected to different swimming regimens allow for a meaningful interpretation of how swimming modulates physiological stress. Previous studies have measured plasma cortisol levels in rainbow trout following between 3 and 24 h of confinement to be 80 ng·ml^−1^ or even higher (Pottinger and Carrick 1999; Trenzado et al. 2003; Kiilerich et al. 2018). In this study, we found that fish in the control group exhibited cortisol levels consistent with values associated with confinement-related stress, while fish subjected to induced swimming showed significantly lower cortisol levels. These findings indicate that short-term swimming activity may attenuate the neuroendocrine stress response under mild confinement, reinforcing the potential of swimming as a modulatory factor for stress physiology.

In the current study, fish subjected to the oscillating swimming condition (O) showed higher plasma lactate when compared to the other groups. Plasma lactate is a by-product and a common indicator of anaerobic metabolism and reflects the reliance on glycolysis during intense physical activity (Weber et al. 2016). In such swimming conditions, fish were subjected to increased energy expenditure, suggesting increased metabolic demand and a shift toward anaerobic energy production due to a change in the gait and the potential appearance of turbulent water flow (Taguchi and Liao, 2011). The elevated levels of plasma lactate after 6 h of swimming at O condition suggest that lactate may be associated with the frequent switch in swimming speeds under the unsteady water current. Under such condition, rainbow trout may possibly recruit an increased proportion of the locomotory muscle when accelerations are involved (Di Santo et al. 2017).

Increased activity of innate immune responses in plasma upon induced swimming has been observed in some freshwater fish species (Zhu et al. 2016; Liu et al. 2018; Hou et al. 2022; Li et al. 2023), while the relation between induced swimming and skin mucus of fish is yet to be investigated. Plasma and skin mucus play distinct but complementary roles in the immune defense of fish. Plasma serves as the transport medium for immune factors, enabling systemic distribution of key immune components (Mokhtar et al. 2023). These factors are crucial for recognizing and neutralizing pathogens throughout the body. However, in the present study, the experimental trials did not trigger any variation in the immune parameters evaluated in plasma. It seems plausible that a longer period and/or different swimming speeds may be needed to be applied in rainbow trout to detect changes in plasma immune parameters as in the studies mentioned above implemented in other species.

The skin mucus acts as the first line of defence at the interface between the fish and its environment, forming a physical and biochemical barrier enriched with antimicrobial enzymes like lysozyme and peroxidase that target pathogens locally (Esteban 2012). Lysozyme and peroxidase activities in skin mucus of rainbow trout were elevated when fish were induced to swim in the L and H conditions compared to the control group. Similarly, a previous study with European eel (*Anguilla anguilla*) showed higher lysozyme and peroxidase activity in skin mucus in fish induced to swim at 0.3 BL s^−1^ for 7 h than the non-swimming group (Espírito-Santo et al. 2024b). Lysozyme plays a crucial role in mediating protection against microbial infections by providing lytic activity against bacterial walls, while peroxidase is involved in oxidative responses against pathogens (Esteban et al. 2013). The elevated activities of lysozyme and peroxidase in the skin mucus of swimming groups may reflect an adaptive response to the induced swimming activity and its associated physiological benefits. This localized immune activation in the skin mucus may allow fish to target potential pathogens at the site of exposure, which is particularly advantageous in animals living in aquatic environments with higher risks of exposure to microbial loads (Esteban 2012). In addition, the physicochemical properties of skin mucus may modify swimming performance by affecting skin friction drag between fish surface and water. A previous study has shown the importance of drag components on the swimming performance of rainbow trout (Yanase et al., 2015); however, the importance that skin mucus characteristics may have on hydrodynamics during swimming remains to be investigated. It has been proposed that increased swimming speed in salmonids causes mucins to aggregate, reducing flow resistance, with the skin mucus acting as a drag-reducing agent (Roberts and Powell 2004). These results highlight the complex interplay between stress and swimming physiology, along with the modulation of the immune response of rainbow trout.

The gene expression analyses showed that swimming conditions had no significant changes in the expression of pro-inflammatory cytokines (*tnfα* and *il1β*), inflammation mediators (*nfκb1*), or immune-related genes (*lyz* and *ctsd*) in the head-kidney, gills, and heart. The swimming regimes applied under the experimental conditions of the current study were insufficient to differently regulate these genes. Similarly, the lack of expression of *nfκb1* gene in the gills and heart, involved in the central regulation of inflammation and stress responses (Ko et al. 2017), suggests that these physiological mechanisms were not triggered in both tissues by the induced swimming applied in the current study. That may indicate that the duration of the swimming activity and the speed applied was within the physiological capacity of rainbow trout and neither compromised immune status nor triggered a detectable inflammatory cascade, as supported by preliminary *U*_*crit*_ measurements performed prior to the trials. With the lack of cytokine induction in the head-kidney, heart, and gill tissues, it can be inferred that the swimming conditions also did not elicit stress-induced inflammation. The absence of a systemic and local inflammatory response is particularly noteworthy, as it indicates that the induced swimming regimes provided physiological stimuli without compromising the immune balance. Interestingly, cathepsin D (*ctsd*) expression was not detected in the gills and heart of fish in the H and O groups but was detected in the C and L groups. Cathepsin D is a lysosomal protease involved in apoptosis and immune regulation, and it has been demonstrated to be down-regulated in the skin of turbot (*Scophthalmus maximus*) subjected to 60 days swimming at 1.8 BL·s^−1^ but not at 0.3 and 0.9 BL·s^−1^ (Li et al. 2019). The absence of *ctsd* expression in the gills and heart of rainbow trout under high-speed and oscillatory swimming conditions may reflect a shift in physiological priorities under increased locomotor demand. Rather than representing a direct energetic saving, this pattern may indicate a temporary down-regulation of immune-related transcription in favor of supporting functions more immediately required during intense or variable swimming activity. While the energetic cost of expressing a single gene such as *ctsd* is likely minimal, overall immune investment may be modulated during periods of elevated muscular activity. Activation of immune response can be energetically costly, as it requires significant metabolic investment in the production of immune cells, cytokines, and other defense molecules (Demas et al. 2012). Under high-speed swimming conditions, where energy demands for locomotion are elevated, it is plausible that energy allocation is prioritized for swimming-related processes over the expression of specific immune-regulatory genes in tissues, such as gills and heart. Other studies support the principle of metabolic trade-offs in fish. In fact, vaccination or immune activation can influence metabolic rates and energy availability in trout, potentially affecting growth performances (Ackerman and Iwama 2000; Skinner et al. 2010). These findings underscore the potential dynamic allocation of energy to physiological processes based on environmental and metabolic demands.

The localized immune modulation observed in the skin mucus, as evidenced by elevated lysozyme and peroxidase activities, along with the lower plasma cortisol levels compared to fish not induced to swim, supports the idea of a controlled immune response during swimming. Such tissue-specific immune modulation may represent an adaptive strategy in fish by balancing localized defense mechanisms while minimizing systemic inflammation (Guo and Dixon 2021). At the same time, avoiding unnecessary systemic inflammation helps preserve energy for other physiological processes, such as swimming, and reduces the risk of tissue damage associated with chronic inflammation. The specific implications of this modulation likely depend on the intensity of the immune challenge and the environmental conditions. Therefore, understanding this balance in the context of different aquaculture settings could help optimize welfare and disease resistance in rainbow trout.

### Energy usage and antioxidant status

In the present study, the activity of CS was increased in red muscle in all induced swimming conditions but not in the control group, while no changes were observed in white muscle. Citrate synthase is responsible for the first step of the Krebs cycle and is widely used as an indicator of aerobic metabolism (Childress and Somero 1979). Thus, increased CS activity in red muscle suggests preeminent aerobic metabolism supporting swimming activity, with energy mainly generated by mitochondria. Conversely, LDH activity, linked to anaerobic metabolism (Negrete et al. 2024), remained unchanged in white muscle, although a significant increase in the O group was observed in red muscle, suggesting the involvement of this type of muscle during the transitions to different swimming speeds. Cytochrome c oxidase activity, which represents the terminal step of the electron transport chain and is critical for ATP production (Negrete et al. 2024), was elevated in the H and O groups compared to both the C and L groups in red muscle. This indicates that a more intense swimming regime may stimulate mitochondrial oxidative phosphorylation in this type of muscle, likely reflecting the higher energy demands associated with these activities. On the other hand, the absence of changes in the activities of CS, LDH, HOAD, and COX in white muscle is consistent with its predominant reliance on anaerobic metabolism during burst swimming (Srinivasan and Avadhani 2012). The lack of changes in HOAD activity between experimental conditions, a key enzyme involved in β-oxidation and fatty acid metabolism (Leaver et al. 2008), suggests that energy provision in white muscle was not significantly supported by oxidative phosphorylation under the swimming conditions tested. This finding reinforces the reliance of white muscle on anaerobic glycolysis for energy generation during burst swimming. Moreover, the unchanged activity of white muscle LDH, an enzyme that facilitates the conversion of pyruvate to lactate in anaerobic metabolism (Torres et al. 2012), may indicate the stability of this pathway in this tissue under all the experimental regimes applied.

Given the limited understanding of how swimming activity influences the antioxidant status in fish and the connection between reactive oxygen species (ROS) and exercise (Birnie-Gauvin et al. 2017; Powers et al. 2020), we examined the impact of different swimming conditions on oxidative stress markers in red and white muscle and liver. Moreover, swimming activity in fish can induce oxidative stress responses due to increased aerobic metabolism at the mitochondrial level (Mortelette et al. 2010). Physical activity-induced oxidative stress is a byproduct of increased metabolic activity and mitochondrial respiration, leading to the generation of ROS (Powers et al. 2020). This is particularly noticeable in fish swimming at increasing intensities, leading to amplified demands for oxygen and metabolic substrate by the contracting muscle (Pengam et al. 2021). Previous studies have shown variable results depending on the species and exercise intensity, making generalizations across species more difficult. Still, recent studies have shown that certain swimming speeds can enhance the antioxidant status in the muscle and liver. A very recent study of Espírito-Santo et al. (2024b) showed that European eel induced to swim at 0.3 BL·s^−1^ for 7 h presented lower LPO levels and higher GSH/GSSG ratio in liver and muscle, compared to fish not induced to swim. Also, it was demonstrated that gilthead seabream (*Sparus aurata*) swimming steadily for 6 h at 0.8 BL·s^−1^ resulted in lower LPO levels in white muscle and increased GSH/GSSG ratio in liver compared to fish not induced to swim or swimming at higher speeds (Espírito-Santo et al. 2024a).

The results of oxidative stress biomarkers observed in the current study revealed changes that can be interpreted as either beneficial or adverse, depending both on the type of swimming activity induced and the type of tissue. High hepatic GST activity in the H group compared to the control group suggests an up-regulation of detoxification pathways to counteract increased ROS production. Glutathione S-transferase is an enzyme involved in the conjugation of glutathione to toxic compounds, highlighting the liver’s central role in controlling oxidative stress (Hellou et al. 2012). Conversely, the increase in hepatic LPO levels observed in the O group suggests that the oscillating swimming regimen enhanced the oxidative stress in this group but not in the control and L groups, possibly due to changes in oxygen and energy demands caused by the fluctuations in swimming speeds. In red muscle, the increased GSH/GSSG ratio in the L and H groups indicates an improvement in redox balance and antioxidant capacity when compared to the other conditions. Elevated GSH levels are associated with enhanced antioxidant capacity, and an increased GSH/GSSG ratio typically reflects a stable cellular redox balance. Glutathione plays a critical role in neutralizing lipid peroxides, converting them into their corresponding alcohols (Aquilano et al. 2014). Additionally, GSH contributes to the removal of peroxides and free radicals by preserving the reduced state of -SH groups in proteins, enzymes, and other molecules (Srikanth et al. 2013). During reactions with oxidants, GSH is transformed into its oxidized form, GSSG, which is then reduced back to GSH by GR. Consequently, elevated GSSG levels are often regarded as an indicator of a stable antioxidant status. This adaptation suggests that sustained swimming enhances red muscle ability to maintain glutathione in its reduced form, thereby buffering against oxidative damage. The absence of changes in LPO levels in red muscle observed further supports the idea that enhanced antioxidant defenses effectively counteract any surge in ROS generated during the swimming conditions applied in the current study. In white muscle, the decreased GSSG levels and the concomitant increase in the GSH/GSSG ratio in the L and H groups reflect a similar enhancement of the antioxidant status as observed in red muscle.

However, under the H condition, a higher involvement of white muscle might be expected compared to the other conditions, as this tissue can contribute to burst-type movements during high-intensity activity (Blake 2004). Furthermore, the O condition and the associated plasma lactate levels observed in this study suggest that white muscle may have actively engaged in anaerobic metabolism in fish subjected to oscillatory swimming. Despite this involvement, the absence of significant changes in oxidative stress responses, such as LPO in white muscle, may be explained by its relatively low mitochondrial and lipid contents, which limits the potential for mitochondrial ROS production and subsequent rise of LPO levels. This is in alignment with the understanding that mitochondria are central in ROS generation and an exceeding production may result in an increase in the oxidative stress of the cells (Powers et al. 2020). Conversely, red muscle is more prone to ROS bursts under increased metabolic activities, due to its higher mitochondrial density and lipid content. This may explain the tissue-specific differences in oxidative stress responses observed in the present study, where LPO levels remained stable in white muscle, but increased in liver, and under oscillatory condition in other tissues. Consequently, the antioxidant adaptations observed in white muscle, such as the increased GSH/GSSG ratio, may reflect systemic effects of swimming-induced up-regulation rather than direct responses to oxidative stress from mitochondrial activity in this tissue.

## Conclusions

This study demonstrates that induced swimming significantly influences stress, immune, and antioxidant responses in rainbow trout. Swimming reduced plasma cortisol levels, indicating a potential to alleviate stress and enhance resilience to environmental challenges. Immune function was enhanced, as shown by elevated lysozyme and peroxidase activities in skin mucus, suggesting improved mucosal immunity and pathogen resistance. Metabolic adaptations were evident in red muscle, with increased CS and COX activity in high-speed and oscillating groups, reflecting enhanced aerobic capacity, while elevated LDH activity in the oscillating group indicated a partial reliance on anaerobic metabolism. White muscle showed no significant metabolic changes, suggesting limited involvement under the swimming conditions applied. Antioxidant defenses were improved in red and white muscle, as indicated by increased GSH/GSSG ratios, though oscillating swimming conditions increased hepatic LPO levels, suggesting a potential risk of oxidative damage. These findings underscore the potential of short-term controlled swimming as a non-invasive tool for beneficial outcomes by improving the response of rainbow trout to programmed events, such as handling or transport, which are stressful to fish. Nevertheless, careful consideration of swimming conditions is necessary to balance benefits against the risk of tissue-specific oxidative stress. Furthermore, as the present work was conducted with individually tested fish over a 6-h period, further studies in group settings and longer durations are required before induced swimming protocols can be recommended for general farm application aiming at long-term beneficial effects. Future research should therefore optimize swimming protocols to maximize fish health, growth, and resilience, ultimately contributing to more sustainable aquaculture systems paired with a clearer understanding of the effects of induced swimming.

## Supplementary Information

Below is the link to the electronic supplementary material.

Supplementary file 1 (DOCX 123 KB)

## Data Availability

No datasets were generated or analysed during the current study.
