# Supplementary material for: Induced swimming reduced stress and modulated immune response and antioxidant status in juvenile rainbow trout (Oncorhynchus mykiss)
Source: Fish Physiol Biochem. 2025 Aug 28;51(5):151. doi: 10.1007/s10695-025-01569-w (PMC12394289; doi:10.1007/s10695-025-01569-w)
Supplement: Supplementary file 1 — (DOCX 123 KB) [file 10695_2025_1569_MOESM1_ESM.docx]

Induced swimming reduced stress and modulated immune response and antioxidant status in juvenile rainbow trout (*Oncorhynchus mykiss*)

Carlos Espírito-Santo^1,2^, Carmen Alburquerque^3^, Thaís Cavalheri^2^, Francisco A. Guardiola^3^, Rodrigo O. A. Ozório^2*^, Leonardo J. Magnoni^4^

^1^ FCUP, Faculty of Sciences, University of Porto, 4169-007 Porto, Portugal.

^2^ CIIMAR/CIMAR-LA, Interdisciplinary Centre of Marine and Environmental Research, University of Porto, Matosinhos 4450-208, Portugal.

^3^ Immunobiology for Aquaculture Group, Department of Cell Biology and Histology, Faculty of Biology, University of Murcia, Murcia 30100, Spain.

^4^ The New Zealand Institute for Plant and Food Research Limited, Nelson 7043, New Zealand.

* Correspondence: rodrigo.ozorio@ciimar.up.pt

**Supplementary Information**

Table S1. Ingredients and proximal composition of the diet.

| Ingredients (%) |  |
| --- | --- |
| Fishmeal | 10.0 |
| Soy protein concentrate | 12.5 |
| Pea protein concentrate | 12.5 |
| Wheat gluten | 13.0 |
| Corn gluten meal | 7.5 |
| Soybean meal | 5.0 |
| Wheat meal | 9.35 |
| Wheat bran | 4.0 |
| Potato starch gelatinized | 6.35 |
| Vit. & Min. Premix | 1.0 |
| Vitamin E | 0.05 |
| Betaine | 0.05 |
| Antioxidant | 0.2 |
| MAP | 1.5 |
| L-Lysine | 0.5 |
| DL-Methionine | 0.2 |
| Silica | 0.4 |
| Fish oil | 3.0 |
| Rapeseed oil | 12.9 |
| Total | 100.0 |
| Diet proximal composition |  |
| Dry matter (DM, %) | 92.0 |
| Crude protein (% DM) | 44.0 |
| Crude fat (% DM) | 18.0 |
| Ash (% DM) | 5.2 |
| Gross energy (MJ kg^-1^) | 21.8 |

~

Table S2. Hematocrit and hemoglobin of rainbow trout (*Oncorhynchus mykiss*) subjected to different swimming conditions for 6 h.

|  | **Experimental conditions** | | | |
| --- | --- | --- | --- | --- |
|  | **C** | **L** | **H** | **O** |
| **Hematocrit** (%) | 28.21 ± 2.59 | 29.51 ± 3.15 | 32.82 ± 2.10 | 31.44 ± 1.95 |
| **Hemoglobin** (g·dL^-1^) | 6.03 ± 0.32 | 5.77 ± 0.45 | 6.94 ± 0.29 | 6.29 ± 0.32 |

L - low-speed (0.8 BL·s⁻¹); H - high-speed (2.3 BL·s⁻¹); O - oscillating speeds (0.8/2.3 BL·s⁻¹) and C – control (< 0.1 BL·s⁻¹). The values represent mean ± SEM (n = 8 fish·treatment^-1^). No significant differences were found between groups (one-way ANOVA, *P* > 0.05).


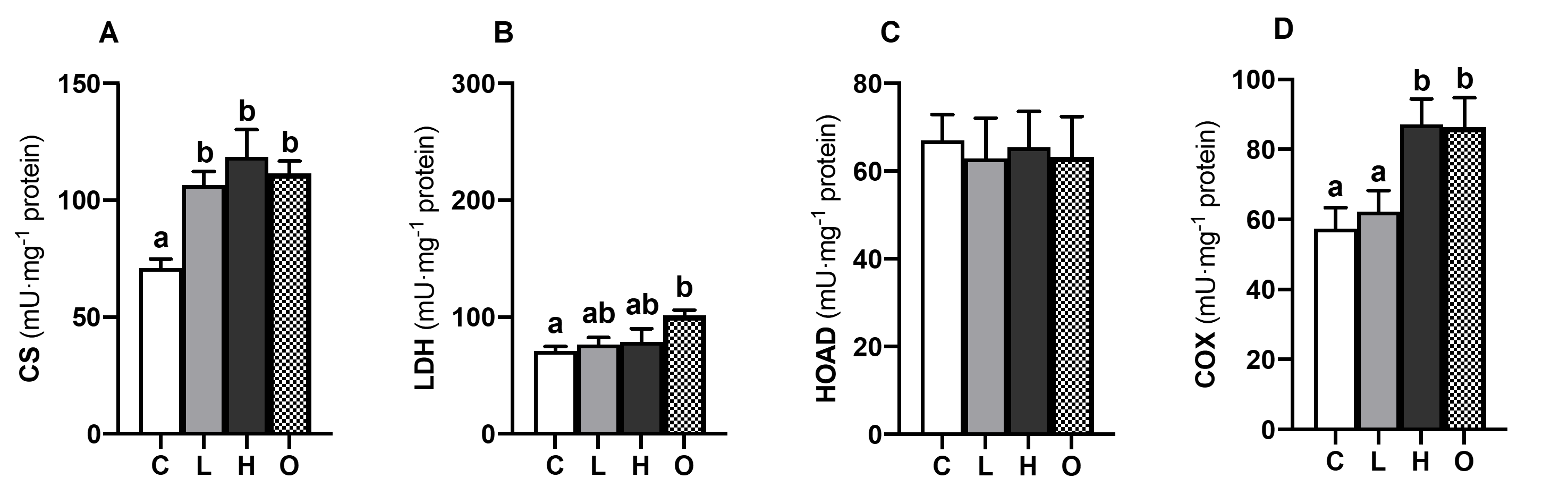


**Fig. S1** Citrate synthase (CS) (A), lactate dehydrogenase (LDH) (B), β-hydroxyacyl CoA dehydrogenase (HOAD) (C), and cytochrome c oxidase (COX) (D) in red muscle of rainbow trout (*Oncorhynchus mykiss*) subjected to different swimming conditions for 6 h: L - low-speed (0.8 BL·s⁻¹); H - high-speed (2.3 BL·s⁻¹); O - oscillating speeds (0.8/2.3 BL·s⁻¹) and C – control (< 0.1 BL·s⁻¹). The values represent mean ± SEM (n = 8 fish·treatment^-1^). Different letters indicate significant differences between the experimental groups (one-way ANOVA, followed by Tukey’s post-hoc test, *P <* 0.05)

**
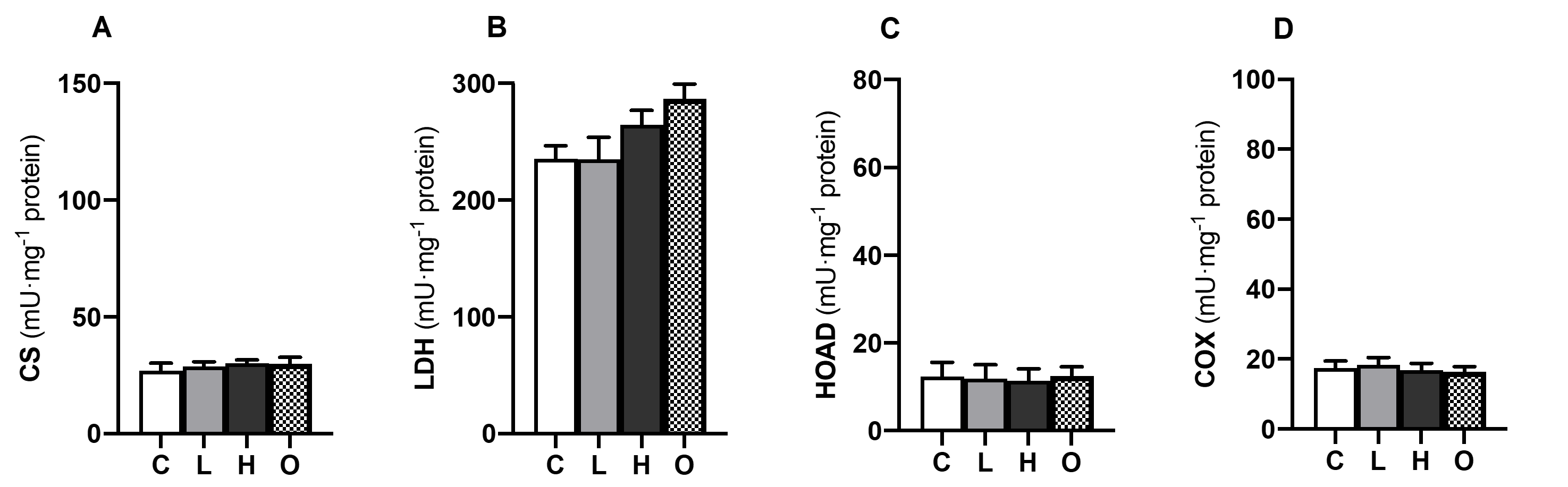
**

**Fig. S2** Citrate synthase (CS) (A), lactate dehydrogenase (LDH) (B), β-hydroxyacyl CoA dehydrogenase (HOAD) (C), and cytochrome c oxidase (COX) (D) in white muscle of rainbow trout (*Oncorhynchus mykiss*) subjected to different swimming conditions for 6 h: L - low-speed (0.8 BL·s⁻¹); H - high-speed (2.3 BL·s⁻¹); O - oscillating speeds (0.8/2.3 BL·s⁻¹) and C – control (< 0.1 BL·s⁻¹). The values represent mean ± SEM (n = 8 fish·treatment^-1^). No significant differences were found between groups (one-way ANOVA, *P* > 0.05)
